# Establishing a Minimum Dataset for Prospective Registration of Systematic Reviews: An International Consultation

**DOI:** 10.1371/journal.pone.0027319

**Published:** 2011-11-16

**Authors:** Alison Booth, Mike Clarke, Davina Ghersi, David Moher, Mark Petticrew, Lesley Stewart

**Affiliations:** 1 Centre for Reviews and Dissemination, University of York, York, United Kingdom; 2 Centre for Public Health, Queen's University Belfast, Belfast, United Kingdom; 3 International Clinical Trials Registry Platform, World Health Organisation, Geneva, Switzerland; 4 Clinical Epidemiology Program, Ottawa Hospital Research Institute, Ottawa, Canada; 5 Department of Epidemiology and Community Medicine, Faculty of Medicine, University of Ottawa, Ottawa, Canada; 6 Department of Social and Environmental Health Research, London School of Hygiene and Tropical Medicine, London, United Kingdom; IUMSP, CHUV/University of Lausanne, Switzerland

## Abstract

**Background:**

In response to growing recognition of the value of prospective registration of systematic review protocols, we planned to develop a web-based open access international register. In order for the register to fulfil its aims of reducing unplanned duplication, reducing publication bias, and providing greater transparency, it was important to ensure the appropriate data were collected. We therefore undertook a consultation process with experts in the field to identify a minimum dataset for registration.

**Methods and Findings:**

A two-round electronic modified Delphi survey design was used. The international panel surveyed included experts from areas relevant to systematic review including commissioners, clinical and academic researchers, methodologists, statisticians, information specialists, journal editors and users of systematic reviews. Direct invitations to participate were sent out to 315 people in the first round and 322 in the second round. Responses to an open invitation to participate were collected separately. There were 194 (143 invited and 51 open) respondents with a 100% completion rate in the first round and 209 (169 invited and 40 open) respondents with a 91% completion rate in the second round. In the second round, 113 (54%) of the participants reported having previously taken part in the first round. Participants were asked to indicate whether a series of potential items should be designated as optional or required registration items, or should not be included in the register. After the second round, a 70% or greater agreement was reached on the designation of 30 of 36 items.

**Conclusions:**

The results of the Delphi exercise have established a dataset of 22 required items for the prospective registration of systematic reviews, and 18 optional items. The dataset captures the key attributes of review design as well as the administrative details necessary for registration.

## Introduction

A protocol should be an integral part of a systematic review, and is important because it pre-specifies the objectives and methods to be used. Having a protocol can help restrict the likelihood of biased post hoc decisions in review methods, such as selective outcome reporting (because it specifies outcomes of primary interest, how information about those outcomes will be extracted, and the methods that might be used to summarize the outcome data quantitatively). An examination of 47 Cochrane reviews revealed indirect evidence for possible selective reporting bias for systematic reviews. Almost all (n = 43) contained a major change, such as the addition or deletion of outcomes, between the protocol and the full publication [1]. However, whether (or to what extent) the changes reflected bias, as opposed to unreported but legitimate changes in methods as the review methods were developed, was not clear. For example, the protocol might have aimed to include specific outcomes, which were then found to be absent from all of the included studies, leading the reviewers to remove these outcomes from their final review. Similarly, setting out inclusion and exclusion criteria prior to author knowledge of the available studies reduces the potential for selective inclusion based on study findings. Publication of a protocol additionally promotes transparency of methods and, as it facilitates identification of reviews that are in process, reduces the potential for unplanned duplication and allows public review of the planned methods.

Capturing the key elements of a systematic review at the protocol stage (or at the design stage if there is no formal protocol) and making these publicly available has similar utility to producing and publishing systematic review protocols. Additionally, a register providing a single point of access should be of great benefit in avoiding unplanned duplication of effort. The issuing of a unique identifier linked to a permanent registration record allows comparison of final reports of reviews with what was planned at registration.

Support for prospective registration of systematic review protocols has been gathering momentum, reflected in a number of recent publications [2], [3], [4], [5]. The PRISMA statement for reporting systematic reviews and meta-analyses of studies that evaluate healthcare interventions advocates registration and the PRISMA 2009 Checklist requires protocol registration details, if available, to include a registration number and details of the existence of and access to the protocol [2], [3].

Until now there has been no widely adopted process to register systematic reviews formally, outside of specific collections of reviews, such as those produced by the Cochrane Collaboration. Recognising the need for registration, the Centre for Reviews and Dissemination (CRD), in collaboration with an international Register Advisory Group, took the initiative in establishing PROSPERO, an international prospective register of systematic reviews with health outcomes that is freely accessible online (www.crd.york.ac.uk/PROSPERO).

The aim of PROSPERO is to prospectively register systematic reviews at the protocol stage; capturing the key attributes of the protocol or plan; maintaining an audit trail of any subsequent protocol amendments; and adding details of final publications, including peer-reviewed articles, and other documents as they become available. This will provide a permanent public record and unbiased listing of registered reviews. PROSPERO can therefore assist in planning new reviews and updating existing ones by providing stakeholders with information about reviews already in the pipeline. This should help to reduce unplanned duplication of effort and to optimise often limited use of research funds.

It will also provide transparency of process, and facilitate comparison between planned methods and reported results enabling readers to make judgements about the importance of any discrepancies [6]. Ultimately this may serve to discourage bias in the conduct and reporting of reviews.

To achieve these aims, the register needs to capture and make available relevant information related to potential for bias in a timely, transparent, and accessible way. At the same time it should be user friendly and not overly burdensome for those completing the registration details. It also needs to be able to accommodate methodological variations between different types of systematic reviews. The development team recognised that support for and use of the register would require the involvement of a range of interested parties including, for example, clinical and academic researchers, commissioners and journal editors. An international consultation was therefore undertaken with the primary objective of establishing the minimum dataset required for registration of systematic reviews at the protocol stage. A secondary objective was to raise awareness of the development of the register.

## Methods

The international Register Advisory Group consists of a small number of key individuals recruited by CRD to assist in taking forward the development of the register. The advisory group members collectively have a wide range of systematic review experience with a variety of methodological interests and significant statistical expertise. In addition members have a detailed knowledge of the Cochrane Collaboration approach to registration of review protocols; experience of clinical trials registers and authorship of the PRISMA statement. The advisory group proposed the use of a Delphi exercise to establish the minimum dataset and subsequently guided each stage of the process.

### Design

A modified Delphi exercise was carried out to obtain opinions from international experts in the field of systematic review about which individual constituents of a review protocol should be included in a registration record. The Delphi technique is a method of collecting in a structured and iterative way, the anonymous, individual opinions of a panel with relevant expertise in the topic where a consensus is required. The basic principle is for the panel to receive successive questionnaires, each one containing the anonymous responses to the previous round, and for them to modify their responses until a consensus is reached [7], [8], [9]. We modified the basic Delphi technique for practical reasons.

The survey population of interest had a high level of Internet and email access, were likely to be familiar with the use of electronic online submission processes and to use email as the principal mode of communication. We aimed to include wide international participation, minimise cost, and ensure accurate and efficient collection and analysis of responses. The questionnaires were therefore administered electronically using on-line survey software Survey Monkey (www.surveymonkey.com).

### Participants

The opinions of international experts in health and social care involved in undertaking, commissioning, or developing methods for systematic reviews, or in guideline development, were sought, as were those of healthcare journal editors.

Two lists of participants were prepared; a core panel of individuals, and an ‘open list’ of organisations, groups, and electronic mailing lists. The initial circulation list for the core panel contained 350 names. These individuals were nominated by members of the register Advisory Group or identified through existing networks (e.g., the PRISMA Group, the International Network of Agencies for Health Technology Assessment; and International Committee of Medical Journal Editors). Email addresses were collected from personal contact lists and publicly available sources (e.g., organisational websites). All emails were personalised to individuals.

The open list included groups such as Guidelines International Network and the Health Technology Assessment International Information Resources Group, for onward dissemination to their members and electronic mailing lists (e.g., Cochrane Methods Groups and the Coordinating Editors of Cochrane Review Groups; LIS-MEDICAL and EVIDENCE-BASED-HEALTH, and World Association of Medical Editors). The open invitation was also posted on websites (e.g., CRD, National Institute for Health Research (NIHR), Cochrane Collaboration, Committee on Publication Ethics) and placed in newsletters (e.g., CRD, Cochrane Collaboration, NIHR). Details of the exercise were published in a Lancet comment paper, which directed readers to the CRD website for further information. This appeared in the e-version of the Lancet during the survey [10] and in the print version at a later date [11].

Separate response collectors were used within Survey Monkey for the two different types of invitation. Anyone responding on a link cascaded by a core panellist would have been included in the core panel collector.

The second round was sent to everyone in the core panel again, including non-responders unless they had requested removal from the list. In addition those from the open list who completed the first round and supplied their email addresses were added to the revised core panel list. Again, separate collectors were used for the core panel and open lists. The second (final) round of the survey required participants to indicate whether they had taken part in the first round. It was accompanied by a summary report on the responses to the first round (available from http://www.york.ac.uk/inst/crd/projects/register.htm).

All responses were anonymous; it was not possible to tell who responded or to link names to responses even when individuals informed us they had responded. It was hoped that this would encourage participation in both rounds and expression of personal opinion, rather than conforming to group opinion or dropping out after the first round [9].

In order to assess representation of different stakeholder groups and identify any differences in the responses between them, simple demographic details were requested in each questionnaire. These were designation; membership of organisations; health area of interest; review method of interest; number of systematic reviews authored; number of systematic reviews in which involved other than as author; proportion of work that relates to methodology; country; and English as a first language.

### Instrumentation

The exercise was limited to two rounds, although provision had been made for subsequent rounds if these were judged necessary by the register Advisory Group. The questionnaires were piloted before distribution.

The time in which the questionnaires were ‘open’ for responses was limited to two weeks for each round. Reminder emails were sent to all members of the core panel approximately one week before the close of each round.

A mixture of ‘pick lists’, pre-specified response options, and free text responses were used to facilitate ease of response and analysis of data from a wide consultation, with large numbers from diverse groups, many of whom may not have English as their first language. In order to ensure that sufficient data were collected and that key areas addressed fully, ‘pick list’ questions were made mandatory. That is respondents had to make a choice before they could submit their answers. It was not mandatory to put anything into the free text boxes.

The questionnaires were prepared by CRD with advice from the register Advisory Group. None of those involved in designing, administering or advising on the questionnaires completed the survey.

The focus for the questions, the language, and explanations used were informed by lessons learned from the development of trials registers, and in particular the requirements for registers as set out by the WHO trials register platform (http://www.who.int/ictrp/en/) [12].

### Question formulation

A pragmatic decision was taken not to approach panellists in advance to ask for their participation. This was to minimise the burden on named individuals who were likely to have limited time to devote to the process. For the same reason, we drew up a list of candidate items for inclusion in the minimum data set based on established guidance for writing systematic review protocols [13], [14], [15], [16], the PRISMA statement [3] and information from the WHO trials registry (http://www.who.int/ictrp/en/).

The first round questionnaire sought preferences for 41 candidate items as to whether they should be included in the minimum data set. Respondents were asked to indicate whether they thought each item was ‘Essential’, ‘Desirable’ or ‘Not necessary’. The focus for responses was on the inclusion of data that would help identify ongoing reviews and enable assessment of bias when the review was completed. Opinions on the scope of the register, allocation of unique ID; timing of registration, dealing with amendments to protocols, publications, and updating of reviews, and existence of other protocol registers were also sought. However, these items relating to the development and implementation of a register are not presented in detail here, but are included in the summary reports, available at http://www.york.ac.uk/inst/crd/projects/register.htm.

The second round questionnaire set out suggestions for which items should be mandatory and which should be optional, based on the register Advisory Group's interpretation of the first round responses. Participants were asked to ‘Agree’, or ‘Disagree’ with the suggested categorisation, to state that an item was ‘Not needed’ or state that they had ‘No opinion’. If they disagreed with a categorisation, they were asked to indicate the direction of the disagreement, e.g., that an item suggested as compulsory should be down-weighted to optional. Again the focus for responses was to identify the minimum dataset to achieve the aims of registration. As with the first round questionnaire, free text boxes for comments and suggestions were provided but not mandatory.

The majority vote for ‘Essential’ or ‘Desirable’ in the first round was used to categorise fields as ‘Required’ or ‘Optional’, respectively for the second round questionnaire.

### Analysis

All responses were collated in ‘Survey Monkey’ for tabulation and analysis. A summary report on each round was compiled and circulated to both distribution lists (available from http://www.york.ac.uk/inst/crd/projects/register.htm).

Where possible, decisions were based on achieving consensus at a designated level of 70% agreement. This level of consensus was agreed by the Advisory Group as being greater than two-thirds of opinion, indicating a clear majority. Other decisions were made taking into consideration the distribution of alternative responses.

### Ethical approval

Formal written consent was not sought; submission of completed questionnaires was taken as implied consent. The research was approved by the University of York Humanities and Social Sciences Ethics Committee (HSSEC 12-2009/10).

## Results

### Responses and respondents

The first round core panel list included 327 direct invitations, 12 were excluded as their emails were returned as undelivered, making the initial list 315. Five people declined to take part and were removed from the mailing list.

The second round core panel list included 322 direct invitations, four were excluded (three emails were returned as undelivered and one was known to be unavailable while the survey was open), making the list 318. One declined to take part and was removed from the mailing list.

A separate collector was set up for the open list invitation to participate. Both the first and second round questionnaires were sent to a general contact at 15 different organisations, and to a named contact for internal circulation in five other organisations or groups.

There were 194 (143 invited and 51 open) respondents with a 100% completion rate in the first round and 209 (169 invited and 40 open) respondents with a 91% completion rate in the second round. Of those who took part in the second round, 113 (54%) said they had taken part in the first round; 72 (34%) said they had not; and 24 (12%) could not remember (Table 1). A comparison of responses to the second round questionnaire showed no significant differences between those taking part in both rounds and those only taking part in the second round.

**Table 1:** Number of responses to questionnaires.

	Number on core panel list	Number who started the survey	Core panel collector	Open collector	Number who completed the survey (%)
First round	315	194	143	51	194 (100)
Second round	318	209	169	40	190 (91)

There were no significant differences between role designations (Table S1); areas of health interest (Table S2); review methods of interest (Table S3); authorship of (Table S4), or involvement in systematic reviews (Table S5); or proportion of work related to research methodology (Table S6); between the first and second round respondents.

There was little difference between the responses of those who were members of The Cochrane Collaboration and those who were not. There were three items in round one and two items in round two where the differences were of statistical significance. After Bonferroni adjustment for multiple comparisons, these were no longer statistically significant (Table S7).

In the first round, 128 (66%) respondents said English was their first language. In the second round, English was the first language for 124 (65%) of respondents. Respondents to both the first and second rounds were based in 34 countries, with an additional six countries represented in the first round only, and a different five countries represented in the second round only (Figure S1).

In the second round we specifically asked participants whether they supported the principle of registration of ongoing systematic reviews; 199 (95.2%) of participants said they did; three (1.4%) did not and seven (3.3%) had no opinion.

### Minimum dataset

Following review of the first round responses, it was decided that the Anticipated publication date field would not be included in the second round. This was because of the large number of comments requesting that the list of items be kept as small as possible, and 158 (82%) respondents felt this field should be optional or was not necessary. The field would be difficult for researchers to estimate at the protocol stage and its inclusion in the register was not integral to achieving the stated aims.

Likewise, 121 (63%) respondents felt it was “Desirable” or “Not necessary” to include the Economic Evaluations field. As this information could and should be included in the Review Question field and elsewhere, it was not included in the second round questionnaire.

Taking into account first round feedback on the need to keep the dataset to the minimum and focus on information that would contribute to reducing bias, it was proposed that although the majority of respondents felt that the Context and Data extraction fields should be required fields, they should be included as optional fields. None of the fields in the first round had a majority in favour of ‘Not needed’.

In the first round of questions, primary and secondary outcomes were presented as separate items from effect measures in order to find out if participants felt both were needed. As only 9% and 12% of the respondents, (respectively for primary and secondary outcomes), felt that effect measures were not necessary, these fields were combined for the second round (Table 2). Time points were added as a requirement in response to suggestions from participants.

**Table 2:** Registration dataset response rates for Delphi round one and two.

	Field title	Delphi first round responses (194)			Delphi second round responses (209)			
		Essential	Desirable	Not necessary	Agree should be Required*/Optional	Disagree should be Optional/Required*	Disagree, not needed	No opinion
1	Review title	174 (90%)	17 (9%)	3 (2%)	189 (98%)*	4 (2%)	0 (0%)	0 (0%)
2	Named contact	186 (96%)	5 (3%)	3 (2%)	187 (97%)*	5 (3%)	0 (0%)	1 (1%)
3	Organisational affiliation of the review	136 (70%)	51 (26%)	7 (4%)	162 (84%)*	23 (12%)	1 (1%)	7 (4%)
4	Named contact address	74 (38%)	91 (47%)	29 (15%)	148 (77%)	30 (16%)*	9 (5%)	6 (3%)
5	Named contact phone number	Item not included in first round			151 (78%)	13 (7%)*	21 (11%)	8 (4%)
6	Named contact email	166 (86%)	26 (13%)	2 (1%)	180 (93%)*	11 (6%)	0 (0%)	2 (1%)
7	Review team	76 (39%)	82 (42%)	36 (19%)	129 (67%)	49 (25%)*	10 (5%)	5 (3%)
8	Review team members' sorganisational affiliations	48 (25%)	104 (54%)	42 (22%)	146 (76%)	27(14%)*	12 (6%)	8 (4%)
9	Collaborators	35 (18%)	106 (55%)	53 (27%)	147 (76%)	18 (9%)*	19 (10%)	9 (5%)
10	Anticipated or actual start date	125 (64%)	57 (29%)	12 (6%)	170 (89%)*	18 (9%)	1 (1%)	3 (2%)
11	Anticipated completion date	91 (47%)	88 (45%)	15 (8%)	152 (79%)*	33 (17%)	3 (2%)	4 (2%)
12	Anticipated publication date	36 (19%)	109 (56%)	49 (25%)	Item not included in second round			
13	Funding sources/sponsors	155 (80%)	31 (16%)	8 (4%)	179 (93%)*	12 (6%)	1 (1%)	0 (0%)
14	Conflicts of interest	152 (78%)	31 (16%)	11 (6%)	173 (90%)*	14 (7%)	3 (2%)	2 (1%)
15	Other registration details	Item not included in first round			134 (70%)	50 (26%)*	8 (4%)	0 (0%)
16	Organisation reference number	55 (28%)	88 (45%)	51 (26%)	139 (72%)	17 (9%)*	18 (9%)	18 (9%)
17	Language	110 (57%)	65 (34%)	19 (10%)	103 (54%)	72 (38%)*	10 (5%)	7 (4%)
18	Country	67 (35%)	83 (43%)	44 (23%)	136 (71%)	33 (17%)*	17 (9%)	6 (3%)
19	Key words	133 (69%)	47 (24%)	14 (7%)	114 (59%)	69 (36%)*	6 (3%)	3 (2%)
20	Any other information	30 (16%)	101 (52%)	63 (33%)	170 (89%)	6 (3%)*	8 (4%)	8 (4%)
21	Review question(s)	186 (96%)	6 (3%)	2 (1%)	186 (97%)*	4 (2%)	1 (1%)	0 (0%)
22	Economic Evaluations	73 (38%)	85 (44%)	36 (19%)	Item not included in second round			
23	Searches	131 (68%)	42 (22%)	21 (11%)	155 (81%)*	32 (17%)	3 (2%)	1 (1%)
24	URL to search strategy	51 (26%)	93 (48%)	50 (26%)	143 (75%)	28 (15%)*	14 (7%)	6 (3%)
25	Types of study to be included	167 (86%)	23 (12%)	4 (2%)	167 (87%)	21 (11%)	3 (2%)	0 (0%)
26	Condition or domain being studied	150 (77%)	35 (18%)	9 (5%)	177 (93%)	11 (6%)	3 (2%)	0 (0%)
27	Participants/population	176 (91%)	14 (7%)	4 (2%)	178 (93%)	12 (6%)	1 (1%)	0 (0%)
28	Intervention(s), exposure(s)	176 (91%)	15 (8%)	3 (2%)	184 (96%)	6 (3%)	1 (1%)	0 (0%)
29	Comparator(s)/control	168 (87%)	24 (12%)	2 (1%)	180 (94%)	9 (5%)	1 (1%)	1 (1%)
30	Context^a^	99 (51%)	77 (40%)	18 (9%)	106 (56%)	77 (40%)	3 (2%)	5 (3%)
31	Primary outcome(s)	180 (93%)	13 (7%)	1 (1%)	177 (93%)	11 (6%)	3 (2%)	0 (0%)
32	Effect measures for primary outcome(s)	126 (65%)	51 (26%)	17 (9%)	(Merged with item 31)			
33	Secondary outcome(s)	130 (67%)	55 (28%)	9 (5%)	146 (76%)	38 (20%)	5 (3%)	2 (1%)
34	Effect measures for secondary outcome(s)	82 (42%)	88 (45%)	24 (12%)	(Merged with item 33)			
35	Data extraction, (selection and coding)^a^	100 (52%)	58 (30%)	36 (19%)	102(53%)	76 (40%)	11 (6%)	2 (1%)
36	Risk of bias (quality) assessment	118 (61%)	54 (28%)	22 (11%)	142 (74%)	35 (18%)	11 (6%)	3 (2%)
37	Strategy for data synthesis	131 (68%)	46 (24%)	17 (9%)	136 (71%)	41(22%)	10 (5%)	4 (2%)
38	Methods for exploring heterogeneity 1^b^	93 (48%)	67 (35%)	34 (18%)	(Merged with 35 and 36 into item 37)			
39	Methods for exploring heterogeneity 2^c^	78 (40%)	76 (40%)	40 (20%)	(Merged with 34 and 36 into item 37)			
40	Definition and rationale for use of specific techniques	73 (38%)	71 (37%)	50 (26%)	(Merged with 34 and 35 into item 37)			
41	Analysis of subgroups or subsets	(Presented in items 34, 35, 36 in first round)			134 (70%)	42 (22%)	10 (5%)	5 (3%)
42	Dissemination plans	35 (18%)	98 (51%)	61 (31%)	151 (79%)	10 (5%)	24 (13%)	6 (3%)
43	Details of any existing review of the same topic by the same authors	139 (72%)	39 (20%)	16 (8%)	124 (65%)	54 (28%)	8 (4%)	5 (3%)

a The majority of respondents in round one selected this as ‘essential’.

b How heterogeneity will be explored. Under what circumstances will a meta-analysis be considered appropriate.

c Covariates to be explored with method of analysis.

Informed by the responses to the Delphi exercise, the register Advisory Group confirmed that all items with 70% or greater agreement would be included as Required or Optional fields as responses indicated.

In round one, there was ≥70% agreement on 14 of 40 items; 60–69% agreement on 7 items; 50–59% agreement on 8 items; 40–49% agreement on 10 items and 30–39% on one item.

After the second round, a 70% or greater agreement was reached on whether 30 of 36 items should be required or optional. There was 60–69% agreement on two and 50–59% agreement on the remaining four items (Table 2).

The final PROSPERO dataset agreed by the register Advisory Group consists of 40 items, 22 of which are required, and the remainder are optional. Of the required fields, 12 are for details of review methods, 10 are related to the review title, timescale and review team (Table 3). In addition, the unique identification number was designated as part of the dataset by the Advisory Group as PROSPERO creates a unique number for each accepted registration record.

**Table 3:** PROSPERO dataset.

**Review title and timescale**		
1	**Review title** *	The working title of the review.
2	**Original language title**	The working title in the language of the review where this is not English.
3	**Anticipated or actual start date** *	The date when the systematic review commenced, or is expected to commence.
4	**Anticipated completion date** *	The date by which the review is expected to be completed.
5	**Stage of review at time of registration** *	The stage of progress of the review at the time of initial registration.
**Review team details**		
6	**Named contact** *	The named contact acts as the guarantor for the accuracy of the information presented in the Register record.
7	**Named contact email** *	The electronic mail address of the named contact.
8	**Named contact address**	The full postal address for the named contact.
9	**Named contact phone number**	The telephone number for the named contact, including international dialling code.
10	**Review team members and their organisational affiliations**	Names of all members of the review team and their organisational affiliations.
11	**Organisational affiliation of the review** *	Details of the organisational affiliations for this review.
12	**Funding sources/sponsors** *	Details of the individuals, organizations, groups or other legal entities who take responsibility for initiating, managing, sponsoring and/or financing the review.
13	**Conflicts of interest** *	Any conditions that could lead to actual or perceived undue influence on judgements concerning the main topic investigated in the review.
14	**Collaborators**	The name, affiliation and role of any individuals or organisations who are working on the review but who are not listed as review team members.
**Review methods**		
15	**Review question(s)** *	The question(s) to be addressed by the review.
16	**Searches** *	Details of the sources to be searched, and any restrictions (e.g. language or publication period).
17	**URL to search strategy**	A link to the search strategy or an example of a search strategy for a specific database.
18	**Condition or domain being studied** *	A short description of the disease, condition or healthcare domain being studied, including health and wellbeing outcomes.
19	**Participants/population** *	Summary criteria for the participants or populations being studied by the review. The preferred format includes details of both inclusion and exclusion criteria.
20	**Intervention(s)/exposure(s)** *	Full and clear descriptions of the nature of the interventions or the exposures to be reviewed.
21	**Comparator(s)/control** *	Details of the alternatives against which the main subject/topic of the review will be compared.
22	**Types of study to be included initially** *	Details of the study designs to be included in the review. If there are no restrictions on the types of study design eligible for inclusion, this should be stated.
23	**Context**	Summary details of the setting and other relevant characteristics which help define the inclusion or exclusion criteria.
24	**Primary outcome(s)** *	The most important outcomes, including information on timing and effect measures, as appropriate.
25	**Secondary outcomes** *	Any additional outcomes that will be addressed, including information on timing and effect measures, as appropriate.
26	**Data extraction (selection and coding)**	The procedure for selecting studies for the review and extracting data, including the number of researchers involved and how discrepancies will be resolved.
27	**Risk of bias (quality) assessment** *	Whether and how risk of bias will be assessed, how the quality of individual studies will be assessed, and whether and how this will influence the planned synthesis.
28	**Strategy for data synthesis** *	The planned general approach to be used, for example whether the data to be used will be aggregate or at the level of individual participants, and whether a quantitative or narrative (descriptive) synthesis is planned.
29	**Analysis of subgroups or subsets** *	Any planned exploration of subgroups or subsets within the review. ‘None planned’ is a valid response if no subgroup analyses are planned.
**General information**		
30	**Type of review**	The type of review.
31	**Language**	The language(s) in which the review is being written and will be made available.
32	**Country**	The country or countries in which the review is being carried out.
33	**Other registration details**	Other places where the systematic review is registered (such as with The Cochrane Collaboration, The Campbell Collaboration, or The Joanna Briggs Institute).
34	**Reference and/or URL for published protocol**	The citation and link for the published protocol, if there is one.
35	**Dissemination plans**	Brief details of plans for communicating essential messages from the review to the appropriate audiences.
36	**Keywords**	The words or phrases that best describe the review.
37	**Details of any existing review of the same topic by the same authors**	Details of earlier versions of the systematic review if an update of an existing review is being registered, including full bibliographic reference if possible.
38	**Review status** *	Indicate the current status of the review.
39	**Any other information**	Any further information the review team consider relevant to the registration.
40	**Link to publication of final report**	The full citation for the final report or publication of the systematic review, including the URL where available.

*Indicates a required field.

## Discussion

Although the drivers for trials registration differ in some respects (e.g., legal ethical requirement [17]), systematic review protocol registration faces the same potential barriers as trials registration. In order to avoid the problems arising from the existence of multiple trials registers [18], [19] by providing a free, single, comprehensive, open access register, a balance between level of detail required and utility was sought. The proposed level of information to be entered for each field was included in the survey as the quality of data recorded in trials registers has been found to vary considerably [20], [21].

The aims of registering a systematic review include the provision of sufficient information to (i) determine whether reviews already in the pipeline might negate the need to initiate a new review, (ii) enhance the transparency and completeness of the plans for the systematic review, and (iii) make informed judgements about potential risk of bias. The objective of this Delphi process was to establish the minimum data set that will achieve these three aims. The Delphi process did not seek to capture the attributes of the wider information that should be included in a full protocol for a systematic review, or to determine all the variables that people might wish to record in registers of systematic reviews that would be used for other purposes.

The Delphi technique was chosen for its flexibility and adaptability in gathering and analysing the necessary data, and in particular for the utility of the process in garnering views and opinions from a broad spectrum of people [8]. The commissioning, undertaking, publishing and use of systematic reviews involves diverse disciplines, each with their own particular perspective, with both inter- and intra-disciplinary differences of opinion. For the register to fulfil its aims and cater for all potential users it was important to ensure that experts from all the relevant disciplines be invited to contribute their opinions in order to reach a consensus. It would not have been possible to arrange face to face meetings with the number of participants achieved by this approach. The Delphi approach allowed us to carry out the consultation with complete anonymity and maintain a broad heterogeneity in participants without any one discipline or individual having more influence than another.

For pragmatic reasons we modified the standard Delphi technique, and discuss here the limitations of the methods we used.

The notion of an ‘international expert’ in the defined areas is largely subjective. We hoped to minimise any inadvertent bias in the selection of the core panel by also issuing an open invitation to participate. However, because of the option of sharing email invitations, we cannot be sure that only core panel members responded to the core panel collector. Nonetheless, a comparison of the data from the two collectors showed little variation in response between the two groups.

Ideally, the same participants should respond to each round of a Delphi process. The pragmatic decision not to approach participants in advance to confirm commitment to the whole exercise, was balanced against the number being invited to take part. Just over half the respondents participated in both rounds. A comparison of second round responses between returning respondents and new participants showed no significant differences. It is unlikely therefore that the approach taken introduced additional bias.

Normally the first round of a Delphi would present open questions such as ‘*What items do you think should be included in the registration of systematic reviews at the protocol stage?*’ However, given that the items that should be included in a systematic review protocol are already well established and to reduce the burden on participants, we invited the first round respondents to comment on the utility of a pre-prepared list of candidate items. Respondents also had the opportunity to suggest additional items. The suggestions that were received and adopted were: the addition of an optional field to record other registration details (e.g., on The Cochrane Library); the requirement of time points to be included in the primary and secondary outcomes fields; and an optional field for telephone contact details.

Based on 315 invitations to participate in the first round, and 143 respondents, the response rate was 45%. In the second round 318 invitations were sent out and 169 responses received, making the response rate 54%. However, the true response rates may be lower as we cannot know how many individuals received a cascaded invitation.

Our decision not to use a pre-determined list of participants for the two rounds was based on the desire to ensure a range of respondents, but could have led to an unrepresentative sample of participants. In the event, responses were received from all key groups and those people who labelled themselves as researchers/reviewers were divided similarly in each round between members (119 round one; 105 round two) and non-members (75 round one; 81 round two) of The Cochrane Collaboration.

We succeeded in gathering the opinions and judgments of a large and diverse range of relevant experts. Given the heterogeneity of the respondents and their interests, we believe that the degree of consensus achieved is acceptable, but we will keep the list of data items under review and will revisit it after it has been in use for a year, as part of a wider evaluation of the utility of PROSPERO.

### Conclusion

The consultation revealed widespread support for the principle of registration of systematic reviews, and the Delphi exercise established a dataset of 22 required items for the prospective registration of systematic reviews, and 18 optional items. The dataset captures the key attributes of review design, as well as the administrative details necessary for registration. The findings were also used to inform the development and implementation of the technical and process elements of PROSPERO.

## Supporting Information

Figure S1 Participant demographic information: Which country are you based in?
(DOC)

Table S1 Professional information about participants: role.
(DOC)

Table S2 Professional information about respondents: health areas of interest.
(DOC)

Table S3 Professional information about respondents: review method of interest.
(DOC)

Table S4 Professional information about respondents: number of systematic reviews authored.
(DOC)

Table S5 Professional information about respondents: number of systematic reviews involved with other than as an author.
(DOC)

Table S6 Professional information about respondents: proportion of work related to research methodology.
(DOC)

Table S7 Professional information about respondents: membership of relevant organisations.
(DOC)
