# Supplementary material for: Establishing a Minimum Dataset for Prospective Registration of Systematic Reviews: An International Consultation
Source: PLoS One. 2011 Nov 16;6(11):e27319. doi: 10.1371/journal.pone.0027319 (PMC3217945; doi:10.1371/journal.pone.0027319)
Supplement: Figure S1 — Participant demographic information: Which country are you based in? (DOC) [file pone.0027319.s001.doc]

**Figure S1.**


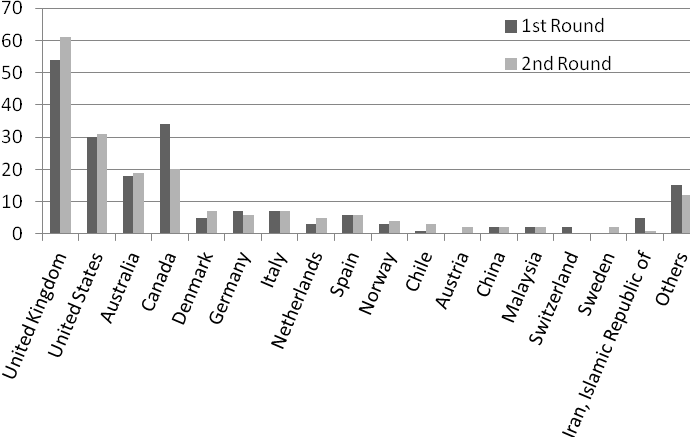


**Participant demographic information: Which country are you based in?**

Other countries are: Bahrain, Finland, Ireland, Republic of Korea, Mexico, Pakistan, Taiwan, (one response in first and second rounds). Columbia, Greece, Hong Kong, India, Israel, New Zealand, (one respondent in first round; none in second round). Argentina, France, Peru, Saudi Arabia, Thailand, (no respondents in first round; one in second round).
