# Supplementary material for: Establishing a Minimum Dataset for Prospective Registration of Systematic Reviews: An International Consultation
Source: PLoS One. 2011 Nov 16;6(11):e27319. doi: 10.1371/journal.pone.0027319 (PMC3217945; doi:10.1371/journal.pone.0027319)
Supplement: Table S1 — Professional information about participants: role. (DOC) [file pone.0027319.s002.doc]

# Table S1. Professional information about respondents: role.

| **Role** | **First round response** | **Second round response** |
| --- | --- | --- |
| Academic clinician | 58 | 53 |
| Clinician | 12 | 10 |
| Commissioner/funder of reviews | 14 | 9 |
| Health economist | 20 | 9 |
| Information specialist | 15 | 27 |
| Journal Editor/board member/involved in publishing | 35 | 44 |
| Researcher (but not a systematic reviewer) | 37 | 25 |
| Statistician | 20 | 12 |
| Systematic reviewer | 110 | 106 |
| Other | 19 | 21 |

N.B. A response to this question was mandatory in the first round: 194 responded. In the second round the question was optional: 190 responded, 19 skipped the question.
