# Supplementary material for: Establishing a Minimum Dataset for Prospective Registration of Systematic Reviews: An International Consultation
Source: PLoS One. 2011 Nov 16;6(11):e27319. doi: 10.1371/journal.pone.0027319 (PMC3217945; doi:10.1371/journal.pone.0027319)
Supplement: Table S2 — Professional information about respondents: health areas of interest. (DOC) [file pone.0027319.s003.doc]

# Table S2. Professional information about respondents: health areas of interest.

| **Health areas** | **First round Response** | **Second round Response** |
| --- | --- | --- |
| Blood and immune system | 16 | 8 |
| Cancer | 35 | 30 |
| Cardiovascular | 22 | 25 |
| Care of the elderly | 11 | 13 |
| Child health | 27 | 28 |
| Complementary therapies | 10 | 9 |
| Dental | 7 | 5 |
| Digestive system | 9 | 11 |
| Ear, nose and throat | 9 | 12 |
| Endocrine and metabolic disorders | 15 | 16 |
| Eye disorders | 8 | 9 |
| Infections and infestations | 24 | 19 |
| Mental health and behavioural conditions | 31 | 25 |
| Musculoskeletal | 25 | 18 |
| Neurological | 19 | 16 |
| Obstetrics and gynaecology | 22 | 17 |
| Oral health | 5 | 8 |
| Perioperative care | 7 | 6 |
| Public health (including social determinants of health) | 75 | 69 |
| Respiratory disorders | 18 | 20 |
| Service delivery | 30 | 23 |
| Skin disorders | 12 | 12 |
| Urological | 11 | 11 |
| Wounds, injuries and accidents | 11 | 13 |
| No specific health area of interest | 53 | 61 |
| Other | 36 | 17 |

N.B. A response to this question was mandatory in the first round: 194 responded. In the second round the question was optional: 190 responded, 19 skipped the question.
