# Supplementary material for: Establishing a Minimum Dataset for Prospective Registration of Systematic Reviews: An International Consultation
Source: PLoS One. 2011 Nov 16;6(11):e27319. doi: 10.1371/journal.pone.0027319 (PMC3217945; doi:10.1371/journal.pone.0027319)
Supplement: Table S3 — Professional information about respondents: review method of interest. (DOC) [file pone.0027319.s004.doc]

# Table S3. Professional information about respondents: review method of interest.

| **Review methods** | **First round response** | **Second round response** |
| --- | --- | --- |
| Effects of health and social care interventions (including rehabilitation and prevention) | 145 | 110 |
| Review methodology | 135 | 132 |
| Reporting of reviews | 78 | 75 |
| Reviews of reviews | 64 | 73 |
| Diagnosis | 66 | 62 |
| Adverse effects | 71 | 58 |
| Qualitative research | 38 | 43 |
| Scoping reviews | 30 | 42 |
| Single technology appraisals | 38 | 39 |
| Economic evaluation | 61 | 39 |
| Prospective meta-analysis | 39 | 36 |
| Screening | 30 | 35 |
| Risk factors | 39 | 32 |
| Prognosis | 27 | 29 |
| Individual participant data | 24 | 19 |
| Study level data | 24 | 17 |
| Genetics | 13 | 14 |
| Other | 17 | 13 |

N.B. A response to this question was mandatory in the first round: 194 responded. In the second round the question was optional: 190 responded, 19 skipped the question.
