# Supplementary material for: Establishing a Minimum Dataset for Prospective Registration of Systematic Reviews: An International Consultation
Source: PLoS One. 2011 Nov 16;6(11):e27319. doi: 10.1371/journal.pone.0027319 (PMC3217945; doi:10.1371/journal.pone.0027319)
Supplement: Table S4 — Professional information about respondents: number of systematic reviews authored. (DOC) [file pone.0027319.s005.doc]

# Table S4. Professional information about respondents: number of systematic reviews authored.

|  | **First round Response** | **Second round Response** |
| --- | --- | --- |
| **0** | 24 | 24 |
| **1-5** | 85 | 67 |
| **6-10** | 30 | 32 |
| **>10** | 55 | 67 |

N.B. A response to this question was mandatory in the first round: 194 responded. In the second round the question was optional: 190 responded, 19 skipped the question.
