# Supplementary material for: Establishing a Minimum Dataset for Prospective Registration of Systematic Reviews: An International Consultation
Source: PLoS One. 2011 Nov 16;6(11):e27319. doi: 10.1371/journal.pone.0027319 (PMC3217945; doi:10.1371/journal.pone.0027319)
Supplement: Table S6 — Professional information about respondents: proportion of work related to research methodology. (DOC) [file pone.0027319.s007.doc]

# Table S6. Professional information about respondents: proportion of work related to research methodology.

|  | **First round Response** | **Second round Response** |
| --- | --- | --- |
| **0** | 10 | 8 |
| **1-40%** | 98 | 110 |
| **41-60%** | 48 | 40 |
| **>60%** | 38 | 32 |

N.B. A response to this question was mandatory in the first round: 194 responded. In the second round the question was optional: 190 responded, 19 skipped the question.
