# Supplementary material for: Establishing a Minimum Dataset for Prospective Registration of Systematic Reviews: An International Consultation
Source: PLoS One. 2011 Nov 16;6(11):e27319. doi: 10.1371/journal.pone.0027319 (PMC3217945; doi:10.1371/journal.pone.0027319)
Supplement: Table S7 — Professional information about respondents: membership of relevant organisations. (DOC) [file pone.0027319.s008.doc]

# Table S7. Professional information about respondents: membership of relevant organisations.

| **Organisation** | **First round response** | **Second round response** |
| --- | --- | --- |
| AHRQ Evidence-based Practice Centers (EPC) Network | 5 | 12 |
| The Campbell Collaboration | 27 | 21 |
| The Cochrane Collaboration | 119 | 105 |
| Committee on Publication Ethics (COPE) | 3 | 7 |
| Council of Science Editors | 1 | 2 |
| Deutsches Netzwerk evidenzbasierte Medizin | 1 | 7 |
| Evaluation of Genomic Applications in Practice and Prevention, U.S. Centers for Disease Control (A working group) | 1 | 1 |
| Guidelines International Network (G-I-N) | 1 | 18 |
| Health Technology Assessment International (HTAi) | 43 | 48 |
| International Committee of Medical Journal Editors (ICMJE) | 2 | 3 |
| International Network of Agencies for Health Technology Assessment (INAHTA) | 2 | 29 |
| International Clinical Epidemiology Network (INCLEN) TRUST | 1 | 4 |
| International Society of Drug Bulletins (ISDB) | 1 | 1 |
| International Society for Pharmacoeconomics and Outcomes Research (ISPOR) | 6 | 9 |
| Partners in Health Technology Assessment (PiHTA) | 1 | 1 |
| Society for Medical Decision Making | 1 | 6 |
| Society for Research Synthesis Methodology (SRSM) | 11 | 10 |
| World Association of Medical Editors (WAME) | 4 | 11 |
| None of these | 41 | 28 |
| Others from 1^st^ round: National Institute for Health and Clinical Excellence (1)  Others from 2^nd^ round: The Joanna Briggs Institute (2); Agency for Healthcare Research and Quality/USPSTF program (1); CILIP (1); CRD advisory Board (1); GRADE member (1); HESG (1); HuGENet (1); Independent Meta-analysis Group (MRC) (1); International Society of Pharmacoepidemiology (1); METCARDIO (www.metcardio.org) (1); Saudi research group (1); Society for Social Medicine (1). |  |  |

N.B. A response to this question was mandatory in the first round: 194 responded. In the second round the question was optional: 190 responded, 19 skipped the question.
